# Cerebral White Matter Lesions on Diffusion-Weighted Images and Delayed Neurological Sequelae after Carbon Monoxide Poisoning: A Prospective Observational Study

**DOI:** 10.3390/diagnostics10090698

**Published:** 2020-09-16

**Authors:** Sangun Nah, Sungwoo Choi, Han Bit Kim, Jungbin Lee, Sun-Uk Lee, Young Hwan Lee, Gi Woon Kim, Sangsoo Han

**Affiliations:** 1 Department of Emergency Medicine, Soonchunhyang University Bucheon Hospital, Bucheon 14584, Korea; potter325@naver.com (S.N.); csw3613@naver.com (S.C.); hanbit6105@gmail.com (H.B.K.); emer0716@naver.com (Y.H.L.); emer0325@naver.com (G.W.K.); 2 Department of Radiology, Soonchunhyang University Bucheon Hospital, Bucheon 14584, Korea; noiese@schmc.ac.kr; 3 Department of Neurology, Korea University Medical Center, Seoul 02841, Korea; furyrage@hanmail.net

**Keywords:** carbon monoxide poisoning, magnetic resonance imaging, neurotoxicity, white matter

## Abstract

Introduction: Carbon monoxide (CO) poisoning can result in delayed neurological sequelae (DNS). Factors predicting DNS are still controversial. This study aims to determine whether acute brain lesions observed using diffusion-weighted magnetic resonance imaging (MRI) following acute CO poisoning are related to the subsequent development of DNS. Methods: This prospective study was conducted on patients with CO poisoning treated at a university hospital in Bucheon, Korea. From August 2016 to July 2019, a total of 283 patients visited the hospital because of CO poisoning. Exclusion criteria included age under 18 years, refusing hyperbaric oxygen therapy, refusing MRI, being discharged against medical advice, being lost to follow-up, having persistent neurological symptoms at discharge, and being transferred from another hospital 24 h after exposure. Results: Of the 154 patients included in the final study, acute brain lesions on MRI (ABLM) were observed in 49 patients (31.8%) and DNS occurred in 30 patients (19.5%). In a logistic regression analysis, lower Glasgow coma scale score and higher exposure time were associated with DNS, and the presence of ABLM in white matter was significantly associated with DNS (OR 6.741; 95% CI, 1.843–24.660; *p* = 0.004). Conclusion: The presence of ABLM in white matter was significantly related to the occurrence of DNS. Early prediction of the risk of developing DNS through MRI may be helpful in treating patients with CO poisoning.

## 1. Introduction

Carbon monoxide (CO) is a colorless, tasteless, and odorless toxic gas produced by incomplete combustion of carbon-based fuel and material [1]. Because of these characteristics, patients often do not know they are affected and, in mild cases, diagnosis and treatment are often delayed because symptoms do not appear immediately. CO has 250 times greater affinity for hemoglobin (Hb) than oxygen, so it binds Hb and reduces oxygen transport. This is a major cause of mortality and morbidity associated with CO poisoning because this process can damage the brain and heart, which are vulnerable to ischemia [2].

DNS is one of the common sequelae of brain damage caused by CO poisoning, which can occur two days to six weeks after successful treatment for acute CO poisoning. DNS should be suspected if symptoms such as memory loss, movement disorder, and/or dementia appear [3]. Previous studies have suggested that old age, loss of consciousness at the time of exposure, and initial neurological abnormality are predictors of DNS following acute CO poisoning, but this remains controversial [4,5]. The predictive values of these factors appear inconsistent because of differences in types of patients, severity of CO poisoning, and diversity of measurement results [6].

Magnetic resonance imaging (MRI) is one of the most accurate tests for diagnosing ischemic brain lesions and brain diseases, such as demyelinating diseases, Alzheimer’s disease, and epilepsy [7,8]. In addition, one of the best ways to present acute ischemic brain lesions is MRI diffusion-weighted images (DWIs) [9,10]. The pathophysiology of CO poisoning results in apoptosis, abnormal inflammatory responses, and ischemia–reperfusion injury in the brain, which can cause ischemic brain tissue damage and demyelination of white matter [3]. Therefore, early ischemic lesions in CO poisoning can be found using MRI, especially DWIs. Previous studies reported that white matter hyperintensities and hippocampal atrophy were usually found on brain MRI in CO poisoning [11,12]. In other studies, the putamen, thalamus, and caudate nucleus were found to be affected, appearing as asymmetric focal hyperintense lesions in T2-weighted and FLAIR images [13,14]. Globus pallidus is also among the regions that are vulnerable to CO [12,15]. Like this, various brain lesions can be found in CO poisoning but there is no consensus on the MRI findings that are related with the development of DNS yet.

This study investigated the association of acute brain lesion on MRI (ABLM), particularly on DWIs, with the occurrence of DNS. The relationship between patterns or locations of ABLMs and the occurrence of DNS was also investigated.

## 2. Methods

### 2.1. Study Design and Setting

This prospective observational study was conducted using a CO registry and telephone interviews with all CO poisoning patients who visited the university hospital emergency department (ED) located in Bucheon, Korea. This study was approved by the hospital institutional trial review board (IRB file No. 2020-03-019).

### 2.2. Participant Selection

**Figure 1:**
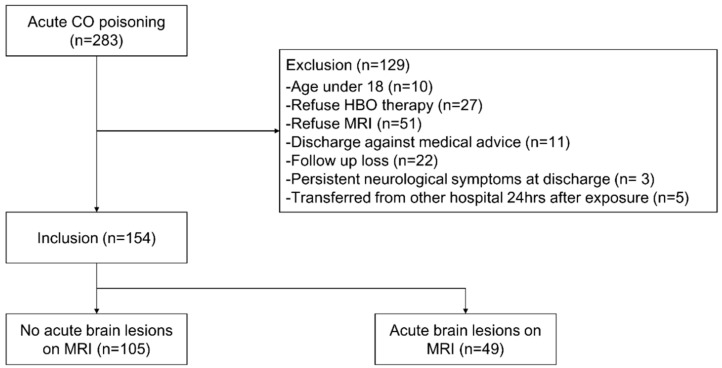
Flow chart of patient selection. Abbreviations: CO, carbon monoxide; HBO, hyperbaric oxygen; MRI, magnetic resonance imaging.

Since August 2016, all patients visiting the ED of our institution with CO poisoning have been placed on the CO registry. All patients who had adequate history or physical examination after CO poisoning, and measured carboxyhemoglobin (COHb) values > 5% in non-smokers and >10% in smokers upon the first visit to the ED were considered to be suffering from CO poisoning. The present study was conducted on CO poisoning patients who visited from August 2016 to July 2019. We included patients who consented to hyperbaric oxygen (HBO) therapy. Patients were excluded if they were under 18 years of age, refused HBO therapy, refused MRI, were discharged against medical advice, were lost to follow-up, had neurological deficits persisting at discharge from the ED, and/or were transferred from another hospital 24 h after exposure (Figure 1). According to our management protocol, patients were scheduled for MRI scans within 2 days of visiting the ED. However, MRI was not performed when patients or their proxy did not consent or when a medical condition contraindicated MRI scan.

### 2.3. HBO Therapy Indication and Protocol

HBO therapy was delivered if the patient’s initial COHb was ≥25% (COHb ≥ 15% in pregnant women), there was a history of loss of consciousness or presence of neurological abnormalities regardless of COHb concentration, and/or there was apparent cardiac injury, such as abnormal electrocardiogram or elevation of troponin I. HBO therapy cannot be applied to endotracheal intubated patients in principle; however, when there were signs of spontaneous breathing and stabilization of vital signs, HBO therapy was delivered. HBO therapy was applied in a monochamber, and treatment was conducted in three sessions at intervals of 6–12 h within a day. In the first session, the total duration of HBO therapy was 150 min/session, and the target pressure was 3 atmospheres (absolute). For later sessions, they were aimed for 120 min/session and 2 atmospheres [16].

### 2.4. Clinical and Laboratory Assessments

At the ED visit, we collected the following data for our registry: demographic data, medical comorbidities, vital signs, Glasgow coma scale (GCS), CO exposure time, intentionality of poisoning, symptoms, and laboratory results. We also prospectively collected neurological symptoms and signs at discharge. DNS were defined as neurological abnormalities that occurred within 3 months of discharge and comprised symptoms such as insomnia, headache, dizziness, Parkinson-like syndrome, movement disorder, conscious disorder, mood disorder, and memory disorder [3,17]. Patients and their proxies were educated about DNS and discharged after receiving relevant documents. Patients visited the ED 1 month after discharge to see an emergency medicine specialist. In addition, telephone interviews about the presentation of DNS were performed with all patients at 2 weeks, and 3 months after discharge. If patients were suspected having DNS symptoms, they were re-admitted and finally diagnosed with DNS through performing additional brain MRI and consulting with neurologists.

### 2.5. Imaging Analysis

MRI examination was performed with a 3-T MRI unit (Signa HDXT 3.0; GE Healthcare, Chicago, IL, USA) using a standard head coil. The DWI parameters were as follows: repetition time, 9000 ms; echo time, 77.5 ms; matrix number, 192 × 192; field of view, 200 mm; 2 b values of 0 and 1000 s/mm^2^; slice thickness, 5 mm; and interslice gap, 1 mm. DWIs and automatically generated apparent diffusion coefficient map were studied (b = 1000 s/mm^2^). DWI was evaluated as follows: (1) presence of pathology, (2) number of pathologies, (3) asymmetry, and (4) location of pathology. Because the size, shape, and distribution of lesions varied, we categorized ABLMs into three patterns: globus pallidus lesions for globus pallidus; diffuse lesions for diffuse symmetric lesions; and focal lesions for asymmetric focal lesions. The presence of ABLM was also categorized by location (cortex, white matter, deep nucleus, brainstem, and cerebellum) and region (frontal, parietal, temporal, occipital, insular, hippocampus, corpus callosum, splenium, internal capsule, centrum semiovale, periventricular white matter, globus pallidus, putamen, caudate, thalamus, and cerebellum) [18]. All images were analyzed under cooperation of radiologist and neurologist while blinded to patient’s information or development of DNS.

### 2.6. Statistical Analysis

Data are presented as absolute numbers or relative frequencies for categorical variables and medians with interquartile ranges for continuous variables. We compared each variable according to the presence of ABLM and DNS. Variables with a *p*-value of <0.05 in univariable analysis were included as candidate variables for multivariable logistic regression modeling. Fisher’s exact test and the chi-squared test were used for categorical variables, while the Mann–Whitney test was used for continuous variables. Adjusted odds ratios (ORs) with 95% confidence intervals (CIs) were also calculated, and two-tailed *p*-values < 0.05 were considered statistically significant. All statistical analyses were performed using SPSS for Windows version 26 (IBM, Armonk, NY, USA).

### 2.7. Modeling

Significant variables were selected using univariable analysis to evaluate the relationship between the occurrence of DNS and the influencing factors including clinical findings and presence of ABLM classified in terms of pattern, region, and location. The presence of ABLM and brain lesions classified by pattern, region, and location have multicollinearity with each other. So, to avoid multicollinearity problems, we used modelling as follows: Model 1, initial GCS, exposure time, and ABLM; Model 2, initial GCS, exposure time, and ABLM classified by pattern; Model 3, initial GCS, exposure time, and ABLM classified by region; Model 4, initial GCS, exposure time, and ABLM classified by location.

## 3. Result

A total of 283 patients visited our ED because of acute CO poisoning. Of these, 129 were excluded for the following reasons: 10 were under 18 years of age, 27 refused HBO therapy, 51 refused MRI, 11 were discharged against medical advice, 22 were lost to follow-up, 3 had persistent neurological symptoms at discharge, and 5 were transferred from another hospital 24 h after exposure (Figure 1).

Thus, a total of 154 patients were included in the study. Of the 154 patients, DNS was observed in 30 patients (19.5%) and the median onset of DNS was 27 days. The DNS group had lower initial GCS score (12.5 vs. 15, *p* = 0.004) and longer CO exposure time (360 vs. 150 min, *p* = 0.072) than the non-DNS group. The DNS group had higher COHb (9.3 vs. 6.0%, *p* = 0.139), C-reactive protein (CRP) (0.23 vs. 0.11 mg/dL, *p* = 0.077), and lactate (2.9 vs. 2.1 mg/dL, *p* = 0.690) than the non-DNS group. The incidence ratio of ABLM in DNS group was higher than that of non-DNS group (60% vs. 25%, *p* = 0.001) (Table 1).

**Figure 2:**
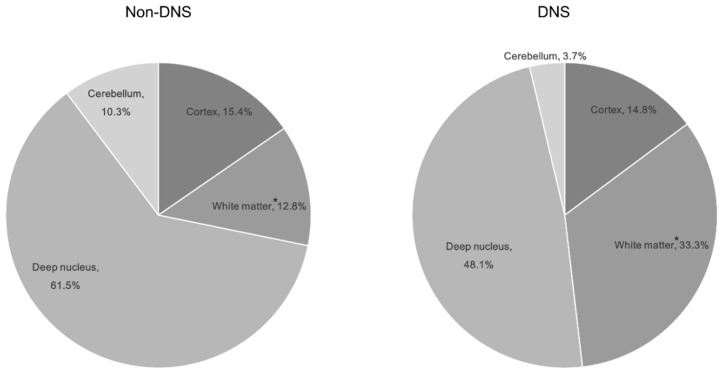
Distribution of brain lesions by location according to the development of delayed neurological sequelae in patients with ABLM. * indicates a significant difference in between the non-DNS and DNS groups (*p* = 0.004). Abbreviations: ABLM, acute brain lesion on magnetic resonance imaging; DNS, delayed neurological sequelae.

All ABLM patterns were significant (*p* < 0.05; Table 2). Significant ABLM regions included the temporal, occipital, and putamen regions (*p* < 0.05; Table 2). Among the locations, only white matter lesions were significant; nine patients with DNS (30%) had a white matter lesion (Table 2, Figure 2).

In the multivariable analysis, four multivariable logistic regression models were used to investigate the significance of categorized brain lesions. The most important variables in each model were as follows: model 1: GCS (OR 0.864; 95% CI, 0.760–0.983; *p* = 0.026), exposure time (OR 1.002; 95% CI, 1.001–1.004; *p* = 0.010), and abnormal MRI (OR 2.242; 95% CI, 0.864–5.821; *p* = 0.097); model 2: GCS (OR 0.882; 95% CI, 0.767–1.014; *p* = 0.078), exposure time (OR 1.002; 95% CI, 1.001–1.004; *p* = 0.006), and diffuse lesion (OR 2.728; 95% CI, 0.948–7.850; *p* = 0.063); model 3: GCS (OR 0.859; 95% CI, 0.755–0.978; *p* = 0.022) and exposure time (OR 1.003; 95% CI, 1.001–1.005; *p* = 0.001); and model 4: GCS (OR 0.849; 95% CI, 0.745–0.967; *p* = 0.014), exposure time (OR 1.003; 95% CI, 1.001–1.004; *p* = 0.005), and white matter (OR 6.741; 95% CI, 1.843–24.660; *p* = 0.004) (Table 3 and Table 4).

## 4. Discussion

There are two types of neurological deficit associated with CO poisoning: persistent neurologic sequelae that occur immediately after exposure and DNS [19]. DNS manifest with symptoms that occur after a lucid interval of approximately 2–40 days, when the patient has already been discharged without symptoms following initial treatment [20]. Because of this clinical course, it is important to predict DNS early to prevent aggravation. Our study revealed that development of subsequent DNS was related to low GCS score, long exposure time, and white matter lesion on MRI; white matter lesion on MRI in particular had a strong association with DNS (OR, 6.741).

### 4.1. Incidence of DNS

DNS occurs in 3–40% of patients with acute CO poisoning [21,22]. DNS is mediated by inflammatory and immune responses, and there is no established diagnostic method to predict this, resulting in differences in reported DNS incidence [6,23]. The DNS incidence rate in this study was 19.48%, consistent with previous studies.

### 4.2. Baseline Characteristics as Predictors for DNS

Factors such as old age, CO exposure time, cardiac arrest, initial neurological abnormalities, GCS, brain imaging abnormalities, and biochemical indicators (e.g., neuron-specific enolase, CRP, lactic acid) are related to DNS following CO poisoning [4,5]. However, it is controversial whether the above factors can serve as predictors of DNS because the outcomes are inconsistent because of the factors such as the types of patients, the severity of CO poisoning, and the diversity of outcome measurements.

Moon et al. reported that GCS is a reasonable predictor of DNS [24]. Weaver et al. also noted that if CO exposure time is long or if there are neurologic symptoms such as loss of consciousness, it is possible to predict the occurrence of DNS [21]. However, it may be impossible or inaccurate to gather an initial GCS or medical history for patients who have attempted suicide through CO poisoning as well as drugs or alcohol, as they may have impaired consciousness [25]. Additionally, patients who attempted suicide are often uncooperative when the medical staff take their history [26]. In our study, 108 (70.13%) patients admitted with CO poisoning were intentionally exposed. To predict DNS, objective factors other than GCS or history taking were needed. Such objective factors may include laboratory findings and brain imaging.

### 4.3. Laboratory Findings as Predictors for DNS

Biochemical indicators such as troponin I and CRP have been studied as factors to predict the occurrence of DNS following CO poisoning [19,27]. Troponin I is a diagnostic tool for assessing myocardial damage in CO poisoning but can also be used as a predictor for DNS because increased troponin I indicates reduced blood supply to the brain, which can lead to neurological abnormalities [27,28]. However, we found that troponin I was not a predictor for DNS, perhaps because it is a component of the contractile apparatus of myocytes and is too specific to myocardial injury compared with other predictors; thus, it may not accurately reflect pathophysiological differences related to neurological results [28]. Pang et al. [29] reported that CRP can be valuable for predicting DNS, but Jung et al. [23] argued that CRP is not related to the development of DNS, and therefore is inappropriate as a predictor for DNS. In our study, laboratory findings such as troponin I and CRP did not correlate with DNS in univariable logistic regression. According to previous report, symptoms caused by CO poisoning were proportional to the level of COHb [30]. However, in our study relatively low level of COHb was measured compared to previous studies, but several symptoms caused by CO poisoning appeared and also DNS occurred [18,19]. Other studies reported that elevated COHb play a fundamental diagnostic role in CO exposure, but it is not correlated with clinical findings and prognosis [6,22]. Because COHb level is affected by factors such as delay from termination of the CO exposure to the time blood is drawn for COHb measurement, as well as intervening administration of oxygen.

### 4.4. Brain Imaging as a Predictor for DNS

Previous studies have explored various imaging tools for predicting DNS following acute CO poisoning but most were found insufficient. Brain lesion on CT scan showed no correlation with the occurrence of DNS in some studies [19,31]. In other studies, electroencephalography and single-photon emission computed tomography (SPECT) were found to be useful in monitoring efficacy of treatment but have failed to predict the development of DNS [32,33]. We investigated whether brain MRI, another brain imaging modality, can play a role in predicting DNS following acute CO poisoning.

Brain lesions caused by CO poisoning can be seen using MRI in both acute and chronic phases because lesions, which are vulnerable to hypoxic damage, appear in the cerebral cortex, white matter, hippocampus, globus pallidus, and cerebellum [13]. Since MRI can diagnose brain damage from CO poisoning, DNS may be more likely to occur in patients with ABLM. Recent studies have proposed that lesions on MRI, especially on DWIs, can predict DNS [18,34]. However, differences in the locations or patterns of MRI lesions have not revealed any correlations with DNS [13]. We found that ABLM was not correlated with the development of DNS in model 1 of multivariable logistic regression.

The main pathological finding of white matter lesions in DNS is demyelination, and MRI shows high signal intensity in white matter [35]. The lesions in white matter destroy the cortex and neurofibrillary network complex that connects the cortex, resulting in multiple cognitive impairments and balance and gait disorders [11,36]. There have been few reports of lesions in white matter during the acute phase. Moon et al. argued that development of DNS is highly likely if a lesion in white matter is found with MRI conducted within 7 days of CO poisoning [24]. Additionally, Jeon et al. reported that MRIs performed within 1 day did not show differences in lesions between DNS and non-DNS groups [18]. In our study, MRI was performed within 2 days of CO poisoning, and as a result, development of DNS was highly associated with white matter ABLMs.

### 4.5. Limitations

There were some limitations to this study. First, because this was a single-center study, it is difficult to generalize the results. A multicenter study is needed to investigate a larger number of patients through a common measurement method. Second, this study determined only the clinical state without using a formal neurocognitive test when evaluating DNS. This may underestimate cognitive impairments that are difficult to detect or may be biased by the physician. Third, this study revealed that white matter lesions are associated with DNS, but it did not reveal how these lesions were related to patient symptoms (e.g., gait disorder, cognitive disorder). Finally, ABLM manifests differently over time based on the injury. In the case of hypoxic damage, a hyperintense signal can be seen on DWIs up to 7 days after the injury [37]. Further research is needed to investigate changes of brain lesions on MRI over time in the context of CO poisoning.

## 5. Conclusions

We found that lower GCS score, higher exposure time, and the presence of ABLM in white matter are predictors of DNS in patients with acute CO poisoning. Since determining GCS and exposure time accurately may be impossible when there is a loss of consciousness, white matter lesions on MRI may be an important objective factor for early prediction of DNS following CO poisoning.

## Figures and Tables

**Table 1:** Comparison of baseline characteristics between the non-DNS and DNS groups.

	Non-DNS (*n* = 124)	DNS (*n* = 30)	*p*-Value
Age, year	40 (29–50.3)	44 (35–55)	0.171
Male, *n* (%)	82 (66.1)	19 (63.3)	0.940
BMI (kg/m^2^)	23.3 (21.0–25.5)	22.8 (21.0–25.4)	0.769
Comorbidities (%)			
Hypertension	9 (7.3)	3 (10.0)	0.902
Diabetes	2 (1.6)	0 (0)	>0.999
Vital signs			
Systolic BP, mmHg	130 (113.5–140)	130 (111.3–143.8)	0.744
Diastolic BP, mmHg	80 (70–90)	80 (70–90)	0.847
Heart rate, BPM	92.5 (78–105)	92 (78.5–100)	0.734
Respiratory rate, BPM	20 (18.8–20)	20 (18–20)	0.215
Body temperature, °C	36.8 (36.5–37.2)	36.9 (36.5–37.5)	0.516
O_2_ saturation, %	98 (97–98)	97.5 (95–98)	0.155
Initial GCS	15 (12–15)	12.5 (6.3–15)	0.004
Exposure time, min	150 (60–315)	360 (90–480)	0.072
Time from exposure to MRI, h	32 (21–48)	43 (24–78)	0.175
Current smoking (%)	60 (50)	15 (51.7)	>0.999
Cause of exposure (%)			0.124
Accidental	41 (33.1)	5 (16.7)	
Intentional	83 (66.9)	25 (83.3)	
Symptoms (%)			
Headache	16 (12.9)	2 (6.7)	0.524
Loss of consciousness	31 (25)	9 (30)	0.743
Chest pain	2 (1.61)	0 (0)	>0.999
Dizziness	16 (12.9)	1 (3.3)	0.239
Dyspnea	5 (4.0)	0 (0)	0.586
Laboratory findings			
COHb, %	6.0 (2.8–11.8)	9.3 (4.2–16.7)	0.139
CRP, mg/dL	0.11 (0.1–0.4)	0.23 (0.1–0.8)	0.077
Lactate, mg/dL	2.1 (1.5–3.9)	2.9 (1.8–4.1)	0.690
Troponin I, ng/mL	0.1 (0.1–0.3)	0.1 (0.1–0.3)	0.942
Ethanol, mg/dL	1.5 (1.0–7.1)	1.2 (0.7–4.2)	0.222
Outcomes			
Onset of DNS, day	-	27 (18–30)	-
ABLM (%)	31 (25)	18 (60)	0.001

Categorical variables are given as numbers (percentage). Continuous nonparametric variables are given as median (interquartile range). Abbreviations: DNS, delayed neurological sequelae; BMI, body mass index; BP, blood pressure; GCS, Glasgow coma scale; MRI, magnetic resonance imaging; COHb, carboxyhemoglobin; CRP, C-reactive protein; ABLM, acute brain lesion on MRI.

**Table 2:** Lesion distribution according to the development of DNS in patients with ABLM.

	No. (%)		
	Non-DNS	DNS	*p*-Value
Pattern			
Globus pallidus lesion	16 (16.2)	11 (36.7)	0.031
Diffuse lesion	13 (13.1)	10 (33.3)	0.024
Focal lesion	5 (5.1)	7 (23.3)	0.007
Region			
Frontal	5 (5.1)	1 (3.3)	>0.999
Parietal	5 (5.1)	3 (10)	0.387
Temporal	2 (2.0)	4 (13.3)	0.026
Occipital	2 (2.0)	4 (13.3)	0.026
Insular	0 (0)	0 (0)	
Hippocampus	3 (3.0)	1 (3.3)	>0.99
Corpus callosum	3 (3.0)	2 (6.7)	0.330
Splenium	2 (2.0)	1 (3.3)	0.551
Internal capsule	1 (1.0)	0 (0)	>0.999
Centrum semiovale	3 (3.0)	1 (3.3)	>0.999
Periventricular white matter	1 (1.0)	2 (6.7)	0.135
Globus pallidus	16 (16.2)	9 (30)	0.157
Putamen	1 (1.0)	3 (10)	0.039
Caudate	1 (1.0)	0 (0)	>0.999
Thalamus	2 (2.0)	1 (3.3)	0.551
Midbrain	0 (0)	0 (0)	
Pons	0 (0)	0 (0)	
Medulla	0 (0)	0 (0)	
Cerebellum	4 (4.0)	1 (3.3)	>0.999
Location			
Cortex	6 (6.1)	4 (13.3)	0.240
White matter	5 (5.1)	9 (30)	<0.001
Deep nucleus	24 (24.2)	13 (43.3)	0.073
Brainstem	0 (0)	0 (0)	
Cerebellum	4 (4.0)	1 (3.3)	>0.999

When multiple lesions appeared in one person, the numbers were aggregated in duplicate. Abbreviations: DNS, delayed neurological sequelae; ABLM, acute brain lesion on magnetic resonance imaging.

**Table 3:** Factors associated with the development of DNS in univariable analysis.

	Univariable Analysis OR (95% CI)
COHb, %	0.984 (0.943–1.027)
Lactate, mg/dL	0.992 (0.730–1.348)
CRP, mg/dL	1.055 (0.964–1.155)
Troponin I, ng/mL	1.164 (0.894–1.516)
Initial GCS	0.853 (0.771–0.944)
Exposure time, min	1.001 (1.000–1.002)
ABLM	3.804 (1.624–8.911)
Globus pallidus (P)	3.003 (1.203–7.501)
Diffuse (P)	3.308 (1.270–8.615)
Focal (P)	5.722(1.664–19.671)
Temporal (R)	7.462 (1.294–43.011)
Occipital (R)	7.462 (1.290–43.010)
Putamen (R)	10.889 (1.089–108.925)
White matter (L)	8.057 (2.448–26.516)

Abbreviations: DNS, delayed neurological sequelae; OR, odds ratio; CI, confidence interval; COHb, carboxyhemoglobin; CRP, C-reactive protein; GCS, Glasgow coma scale; ABLM, acute brain lesion on MRI; P, pattern; R, region; L, location.

**Table 4:** Factors associated with the development of DNS in multivariable analysis.

	Multivariable Analysis			
	Model 1 OR (95% CI)	Model 2 OR (95% CI)	Model 3 OR (95% CI)	Model 4 OR (95% CI)
Initial GCS	0.864 (0.760–0.983)	0.882 (0.767–1.014)	0.859 (0.755–0.978)	0.849 (0.745–0.967)
Exposure time, min	1.002 (1.001–1.004)	1.003 (1.001–1.004)	1.003 (1.001–1.005)	1.003 (1.001–1.004)
ABLM	2.242 (0.864–5.821)			
Globus pallidus (P)		1.160 (0.364–3.695)		
Diffuse (P)		2.728 (0.948–7.850)		
Focal (P)		2.564 (0.527–12.477)		
Temporal (R)			1.229 (0.109–13.868)	
Occipital (R)			5.643 (0.440–72.543)	
Putamen (R)			2.223 (0.115–43.033)	
White matter (L)				6.741 (1.843–24.660)

Abbreviations: DNS, delayed neurological sequelae; OR, odds ratio; CI, confidence interval; GCS, Glasgow coma scale; ABLM, acute brain lesion on MRI; P, pattern; R, region; L, location.
